# Circ_DOCK1 regulates USP11 through miR-132-3p to control colorectal cancer progression

**DOI:** 10.1186/s12957-021-02173-x

**Published:** 2021-03-08

**Authors:** Weitong Zhang, Zhenfen Wang, Guohao Cai, Ping Huang

**Affiliations:** Department of Anorectal Surgery, Hainan General Hospital, No. 19 Xiuhua Road, Haikou, 570311 Hainan China

**Keywords:** Colorectal cancer, circ_DOCK1, miR-132-3p, USP11

## Abstract

**Background:**

Circular RNAs (circRNAs) take part in colorectal cancer malignancies. CircRNA dedicator of cytokinesis 1 (circ_DOCK1) is involved in colorectal cancer progression, but the mechanism underlying this circRNA that takes part in colorectal cancer development remains largely undetermined.

**Methods:**

Tumor and normal para-cancerous tissues were collected from 42 colorectal cancer patients. Human colorectal cancer cell lines (HCT116 and SW480) were used for the experiments in vitro. Circ_DOCK1, microRNA (miR)-132-3p, and ubiquitin-specific protease 11 (USP11) levels were measured through quantitative real-time polymerase chain reaction and Western blotting. Cell growth, metastasis, and apoptosis were investigated via colony formation, 5-ethynyl-2′-deoxyuridine (EdU) staining, MTT, flow cytometry, Western blotting, and transwell analyses. The target association was evaluated via dual-luciferase reporter analysis, RNA pull-down, and immunoprecipitation (RIP). Xenograft assay was performed using HCT116 cells. USP11 and Ki67 levels in tumor tissues were detected via immunohistochemistry.

**Results:**

Circ_DOCK1 expression was enhanced in colorectal cancer tissues and cells. Silencing circ_DOCK1 repressed cell growth, migration, and invasion, and facilitated apoptosis. Circ_DOCK1 sponged miR-132-3p, and miR-132-3p silence mitigated the effect of circ_DOCK1 interference on cell growth, metastasis, and apoptosis. MiR-132-3p targeted USP11, and circ_DOCK1 could regulate USP11 level by miR-132-3p. MiR-132-3p suppressed cell growth, metastasis, and apoptosis, and USP11 attenuated these effects. Knockdown of circ_DOCK1 decreased colorectal cancer cell xenograft tumor growth.

**Conclusion:**

Circ_DOCK1 interference suppressed cell growth and metastasis, and increased apoptosis of colorectal cancer via decreasing USP11 by increasing miR-132-3p.

**Supplementary Information:**

The online version contains supplementary material available at 10.1186/s12957-021-02173-x.

## Highlights

1. Circ_DOCK1 expression is increased in colorectal cancer.
2. Circ_DOCK1 interference inhibits cell growth and metastasis, and induces apoptosis.
3. Circ_DOCK1 regulates USP11 by sponging miR-132-3p.
4. Circ_DOCK1 controls colorectal cancer development via modulating miR-132-3p and USP11.
5. Circ_DOCK1 knockdown decreases tumor growth.

## Introduction

Colorectal cancer is a deadly cancer accounting for about 10% of all diagnosed cancers and cancer-related death worldwide [1]. The incidence has a decreasing trend in highly developed countries, while it is predictive to increase to 2–5 million new cases in 2035 [1]. About 20–30% patients are diagnosed at advanced stages, and 40–50% of those at early stages develop relapse [2]. The 5-year survival rate for patients at early stages is 90%, and the survival rate for those at advanced stages is 13.1% [3]. With the advance in the diagnosis and treatment, great improvement has been gained on the prognosis of patients with early colorectal cancer [4]. However, the survival of patients with the advanced malignancy remains low. Hence, there is a need to explore new mechanism for understanding colorectal cancer pathogenesis and find novel strategy for colorectal cancer therapy.

Noncoding RNAs are involved in cancer phenotypes, and noncoding RNA-based intervention may provide new landscapes for colorectal cancer treatment [5]. Circular RNAs (circRNAs) are a type of noncoding RNA molecules related to various processes like proliferation, apoptosis, migration, and invasion by modulating microRNAs (miRNAs) and mRNAs in colorectal cancer [6]. For instance, hsa_circ_0026416 facilitates cell proliferation and migration by regulating miR-346 and Nuclear Factor I/B (NFIB) in colorectal cancer [7]. Moreover, hsa_circ_0106714 can repress colorectal cancer cell proliferation, migration, and invasion by regulating miR-942-3p/discs large homolog 2 (DLG2) axis [8]. The circRNA dedicator of cytokinesis 1 (circ_DOCK1) has multiple circRNA isoforms, like has_circ_100721, hsa_circ_0020394, hsa_circ_0007142, and hsa_circ_0020397 [9–12]. More importantly, hsa_circ_0007142 and hsa_circ_0020397 are upregulated and have important roles in colorectal cancer [11, 12]. Our study focused on hsa_circ_0020397 isoform. Nevertheless, the regulatory networks of circ_DOCK1 (hsa_circ_0020397) are complex, and additional axis should be explored.

MiRNAs are small noncoding RNAs that are associated with the diagnosis, development, and therapy of colorectal cancer [13]. Multiple evidences suggest miR-132-3p can serve as a tumor suppressor via regulating various mRNAs in human tumors, like lung adenocarcinoma, retinoblastoma, and liver cancer [14–16]. Moreover, the downregulated miR-132-3p predicts the worse prognosis of colorectal cancer [17], and miR-132-3p suppresses colorectal cancer metastasis via regulating zinc-finger E-box-binding homeobox-2 (ZEB2) [18]. A previous study suggests circ_DOCK1 (hsa_circ_0020394) can sponge with miR-132-3p in bladder carcinoma [10]. Yet no study reports miR-132-3p is associated with circ_DOCK1 (hsa_circ_0020397)-mediated network in colorectal cancer. Protein ubiquitination is a classic post-translational modification and involved in multiple cell processes [19, 20]. The ubiquitination controls many processes to participate in the development of diseases, and modulating ubiquitination might be helpful for providing strategy for the therapy of cancers [21]. Ubiquitin-specific protease 11 (USP11) is an important deubiquitinase [22]. More importantly, the emerging evidence suggests that USP11 can promote the development of colorectal cancer by protecting protein phosphatase 1 via deubiquitination to activate mitogen-activated protein kinase pathway [23]. We performed the bioinformatic analysis to predict the potential interaction between miR-132-3p and circ_DOCK1 or USP11. Hence, we hypothesized the circ_DOCK1/miR-132-3p/USP11 axis might be an important network for circ_DOCK1 in colorectal cancer.

In this research, the purposes were to investigate the function of circ_DOCK1 (hsa_circ_0020397) on cell growth, metastasis, and apoptosis in colorectal cancer, and to explore whether it required the circ_DOCK1/miR-132-3p/USP11 network.

## Materials and methods

### Bioinformatic analysis

The binding sites of miR-132-3p and circ_DOCK1 were predicted via circBank (http://www.circbank.cn/), a comprehensive database of human circRNA predicting the miRNA-circRNA interactions [24]. The binding sites of miR-132-3p and USP11 3′ UTR were predicted by starBase (http://starbase.sysu.edu.cn/), a platform for studying the miRNA-mRNA interactions [25].

### Clinical tissues

Forty-two colorectal cancer patients were listed from Hainan General Hospital. The tumor and normal para-cancerous tissues were obtained from same patients by surgical procedures and maintained in liquid nitrogen. All subjects have signed the written informed consents, and this study was permitted via the Ethics Committee of Hainan General Hospital, and performed in line with the Declaration of Helsinki.

### Cell culture

Human colorectal cancer cell lines (HCT116 and SW480) and control fetal colon cell line (FHC) were provided via American Type Culture Collection (ATCC, Manassas, VA, USA) and grown in Dulbecco’s Modified Eagle Medium (DMEM; Thermo Fisher, Wilmington, DE, USA) plus 10% fetal bovine serum (Gibco, Gran Island, NY, USA) and 1% penicillin-streptomycin (Gibco) at 37 °C with 5% CO_2_.

### Quantitative real-time polymerase chain reaction (qRT-PCR)

Total RNA was isolated with Trizol (Thermo Fisher) according to the protocols. The RNA in nucleus or cytoplasm was prepared with Cytoplasmic & Nuclear RNA Purification kit (Norgen Biotek, Thorold, Canada) according to the manufacturer’s instructions. Five hundred nanograms of RNA was used for cDNA synthesis through TaqMan cDNA synthesis kit (Thermo Fisher) following the protocols. The synthesized cDNA was mixed with SYBR (Solarbio, Beijing, China) and primer pairs for qRT-PCR on ABI 7500 FAST Real-Time PCR System (Applied Biosystems, Foster City, CA, USA). The primers were synthesized from Sangon (Shanghai, China) and shown in Table 1. U6 (for miRNAs or nucleus) or β-actin (for circRNAs, mRNAs, or cytoplasm) served as endogenous reference. Relative RNA level was analyzed with 2^−ΔΔCt^ [26].

**Table 1:** The primer sequences for qRT-PCR in this study

Name	Sequence (5′-3′)	
	Forward	Reverse
miR-132-3p	GCCGAGTAACAGTCTACAGCC	AGTGCAGGGTCCGAGGTATT
U6	TTCCTCCGCAAGGATGACACGC	GTGCAGGGTCCGAGGT
circ_DOCK1	CATTTCCATGGGACGAGATT	CCTCGGTACCACCCTTCATA
DOCK1	TATGAAGGGTGGTACCGAGG	TAGAGCTGCCTCCAGATGGT
USP11	TTCCACGGCCTCTTCAAGTC	CGCGGATCCATGGGGATAAA
GAPDH	AATGGGCAGCCGTTAGGAAA	GCGCCCAATACGACCAAATC

### Circular structure analysis

The circular structure of circ_DOCK1 was analyzed via actinomycin D assay. Briefly, HCT116 and SW480 cells were exposed to 2 μg/mL actinomycin D (Glpbio, Montclair, CA, USA) for 0, 8, 16, or 24 h. Then, cells were harvested for detection of circ_DOCK1 and DOCK1 mRNA levels by qRT-PCR as mentioned above.

### Cell transfection

USP11 overexpression vector was formed by cloning in pcDNA3.1 vector (Thermo Fisher), and vector alone was used as control (pcDNA). The small interfering RNA (siRNA) for circ_DOCK1 (si-circ_DOCK1), siRNA negative control (si-NC), miR-132-3p mimic, mimic negative control (miR-NC), miR-132-3p inhibitor (anti-miR-132-3p), and inhibitor negative control (anti-miR-NC) were synthesized via RiboBio (Guangzhou, China), and the oligo sequences are shown in Table 2. For cell transfection, 1 μg of constructed vector or 30 nM of oligonucleotides (siRNAs or miRs), and 10 μL Lipofectamine™ 2000 (Thermo Fisher) were diluted in 250 μL Opti-MEM (Thermo Fisher). HCT116 and SW480 cells were incubated with the mixture for 6 h, and then cultured with complete medium for 24 h.

**Table 2:** The oligo sequences for transfection in this study

Name	Sequence (5′-3′)
si-circ_DOCK1	UAGUUAUAAAAAGAAUGAGUU
si-NC	AUCAAUAUUUUUGAAUGAGUU
miR-132-3p mimic	UAACAGUCUACAGCCAUGGUCG
miR-NC	CGAUCGCAUCAGCAUCGAUUGC
anti-miR-132-3p	GCACCAUGGCUGUAGACUGUUA
anti-miR-NC	UGAGCUGCAUAGAGUAGUGAUUA

### Colony formation assay

HCT116 and SW480 cells (600 cells/well) were dispersed in 6-well plates and incubated for 12 days. Then, cells were stained using 0.5% crystal violet (Solarbio) for 10 min. Next, the colonies were imaged and counted.

### 5-Ethynyl-2′-deoxyuridine (EdU) staining

Cell proliferative ability was investigated using BeyoClick™ EdU Cell Proliferation Kit with Alexa Fluor 488 (Beyotime, Shanghai) following the instructions as a previous report [27]. In brief, 4 × 10^4^ HCT116 and SW480 cells were seeded in 24-well plates and cultured for 24 h. Then, cells were nurtured with 10 μM EdU for 2 h, followed by fixing with 4% paraformaldehyde (Beyotime) for 15 min and treatment of 0.3% Triton X-100 (Beyotime) for 10 min. Next, cells were incubated with the click reaction solution for 30 min. The nuclei were stained with Hoechst 33342 for 10 min. Cells were observed with a fluorescence microscope (Olympus, Tokyo, Japan). The ratio of EdU-positive cells (EdU-positive cells/Hoechst 33342-stained cells) was calculated to investigate the proliferative ability.

### 3-(4,5-dimethylthiazol-2-yl)-2,5-diphenyl-tetrazolium bromide (MTT)

HCT116 and SW480 cells (4 × 10^3^ cells per well) were placed in 96-well plates in triplicate. After the incubation at 37 °C for 0, 1, 2, and 3 days, 0.5 mg/mL MTT solution (Beyotime) was infused. Cells were incubated for 4 h, and then, medium was removed. Next, 200 μL of DMSO (Solarbio) was utilized to dissolve the formed formazan. The optical density (OD) value was examined at 570 nm through a microplate reader (Bio-Rad, Hercules, CA, USA).

### Flow cytometry

For cell cycle distribution analysis, 1 × 10^5^ HCT116 and SW480 cells were dispersed in 12-well plates and incubated for 3 days. After fixture using 75% ethanol (Aladdin, Shanghai, China), cells were dyed with propidium iodide (PI) (Beyotime). Cycle process was detected with a flow cytometer (Agilent, Beijing, China).

Annexin V-fluorescein isothiocyanate (FITC) apoptosis detection kit (Beyotime) was used for detection of cell apoptosis. 1 × 10^5^ HCT116 and SW480 cells were added in 12-well plates in triplicate, and then incubated at 37 °C for 3 days. Then, cells were resuspended in Annexin V binding buffer (50 mM HEPES, 700 mM sodium chloride, 12.5 mM calcium chloride, and 5% bovine serum albumin), followed by staining with Annexin V-FITC and PI. The apoptotic cells were examined with a flow cytometer.

### Transwell assay

Cell migration and invasion were examined in 24-well plates with transwell inserts (Corning, Corning, NY, USA). For migration analysis, HCT116 and SW480 cells (3 × 10^4^ cells per well) in serum-free DMEM were placed in the upper chambers. For invasion analysis, the inserts were pre-coated with Matrigel, and 1 × 10^5^ cells in serum-free medium were added in the upper chambers. The lower chambers were filled with DMEM plus 10% fetal bovine serum. After the incubation for 24 h, cells in the lower chambers were stained using 0.1% crystal violet, and imaged with a × 100 magnification microscope (Olympus).

### Western blotting

The total protein was extracted from tissue homogenate or cell lysate via the RIPA buffer (Beyotime). After the centrifugation, the supernatant was harvested and quantified through BCA protein assay kit (Beyotime) according to the manufacturer’s instructions. Next, 20 μg of protein was separated via 10% sodium dodecyl sulfate-polyacrylamide gel electrophoresis, and transferred to polyvinylidene difluoride membranes (Bio-Rad). The membranes were blocked in the blocking buffer (Beyotime) for 1 h, and then incubated with primary antibodies for anti-CyclinD1 (ab226977, 1:300 dilution, Abcam, Cambridge, UK), anti-USP11 (ab109232, 1:3000 dilution, Abcam), or anti-β-actin (ab8227, 1:3000 dilution, Abcam) overnight, followed by incubation of horseradish peroxidase (HRP)-labeled IgG (ab6721, 1:10000 dilution, Abcam) for 2 h. β-actin was used as a loading control. The protein signaling was developed via Enhanced Chemiluminescence (Solarbio). Relative protein level was analyzed using QuantityOne v4.6 (Bio-Rad) and normalized to GAPDH.

### Dual-luciferase reporter, RNA pull-down, and immunoprecipitation (RIP) assays

The wild-type (WT) sequence of circ_DOCK1 or USP11 with miR-132-3p binding sites was cloned in pGL3 vector (Promega, Madison, WI, USA), forming the circ_DOCK1-WT and USP11-WT luciferase reporter vectors. The mutant (MUT) luciferase reporter vectors (circ_DOCK1-WT and USP11-MUT) were constructed using the mutated sequences. The constructed vectors and miR-NC or miR-132-3p mimic were transfected in HCT116 and SW480 cells for 24 h. Luciferase activity was examined with a dual-luciferase assay kit (Promega).

A Pierce™ Magnetic RNA-Protein Pull-Down kit (Thermo Fisher) was used to analyze the binding of circ_DOCK1 and miR-132-3p following the instructions. In brief, the biotin-labeled circ_DOCK1-WT (Bio-circ_DOCK1-WT), Bio-circ_DOCK1-MUT, or negative control (Bio-NC) was generated through the RNA 3’ end desthiobiotinylation kit (Thermo Fisher) and incubated with the streptavidin magnetic beads and lysates of HCT116 and SW480 cells overnight. The enriched miR-132 level was measured via qRT-PCR.

A RIP kit (Geneseed, Guangzhou, China) was used to analyze the binding of miR-132-3p and USP11. In brief, 1 × 10^7^ HCT116 and SW480 cells were lysed using RIP lysis buffer, and incubated with beads pre-coated with anti-Ago2 (ab186733, 1:50 dilution, Abcam), or anti-IgG (ab190475, 1:100 dilution, Abcam) overnight. The enrichment levels of miR-132-3p and USP11 were examined via qRT-PCR.

### Xenograft model

The animal research was approved via the ethics committee of Hainan General Hospital and was processed following the experimental animal use guidelines of the National Institutes of Health. The lentivirus vectors of shRNA against circ_DOCK1 (sh-circ_DOCK1) or negative control (sh-NC) were formed via GeneChem (Shanghai, China), and transfected in HCT116 cells. Five-week-old male BALB/c nude mice were obtained from Charles River (Beijing, China) and subcutaneously injected with 2 × 10^6^ sh-circ_DOCK1- or sh-NC-transfected HCT116 cells (*n*=5). The tumor volume was examined weekly after cell injection for 10 days and calculated via 0.5 × length × width^2^. After 30 days, the mice were killed via inhalation anesthesia of 5% isoflurane (Sigma), and tumor weight was measured. The levels of circ_DOCK1, miR-132-3p, USP11, and Ki67 in tumor tissues were measured via qRT-PCR, Western blotting, or immunohistochemistry.

### Immunohistochemistry

The tumor tissues were fixed with 4% paraformaldehyde, dehydrated and embedded in paraffin, followed via cutting in 4-μm sections. Next, the sections were rehydrated and blocked using 3% H_2_O_2_. Then, sections were incubated with primary antibodies for USP11 (ab247005, 1:200 dilution, Abcam) or Ki67 (ab15580, 1:500 dilution, Abcam) for 12 h and HRP-labeled IgG (ab6721, 1:1000 dilution, Abcam) for 2 h. After the diaminobenzidine (DAB; Beyotime) staining, sections were observed under a microscope.

### Statistical analysis

The graphic and statistical analysis were performed with GraphPad Prism 6 (GraphPad Inc., La Jolla, CA, USA). The experiments were repeated three times with 3 technical replicates, unless otherwise indicated. The results were expressed as mean ± standard deviation (SD). The linear correlation was investigated by Pearson test. The comparison was conducted using Student’s *t*-test or ANOVA, and it was significant at *P*<0.05.

## Results

### Circ_DOCK1 is upregulated in colorectal cancer

**Fig. 1:**
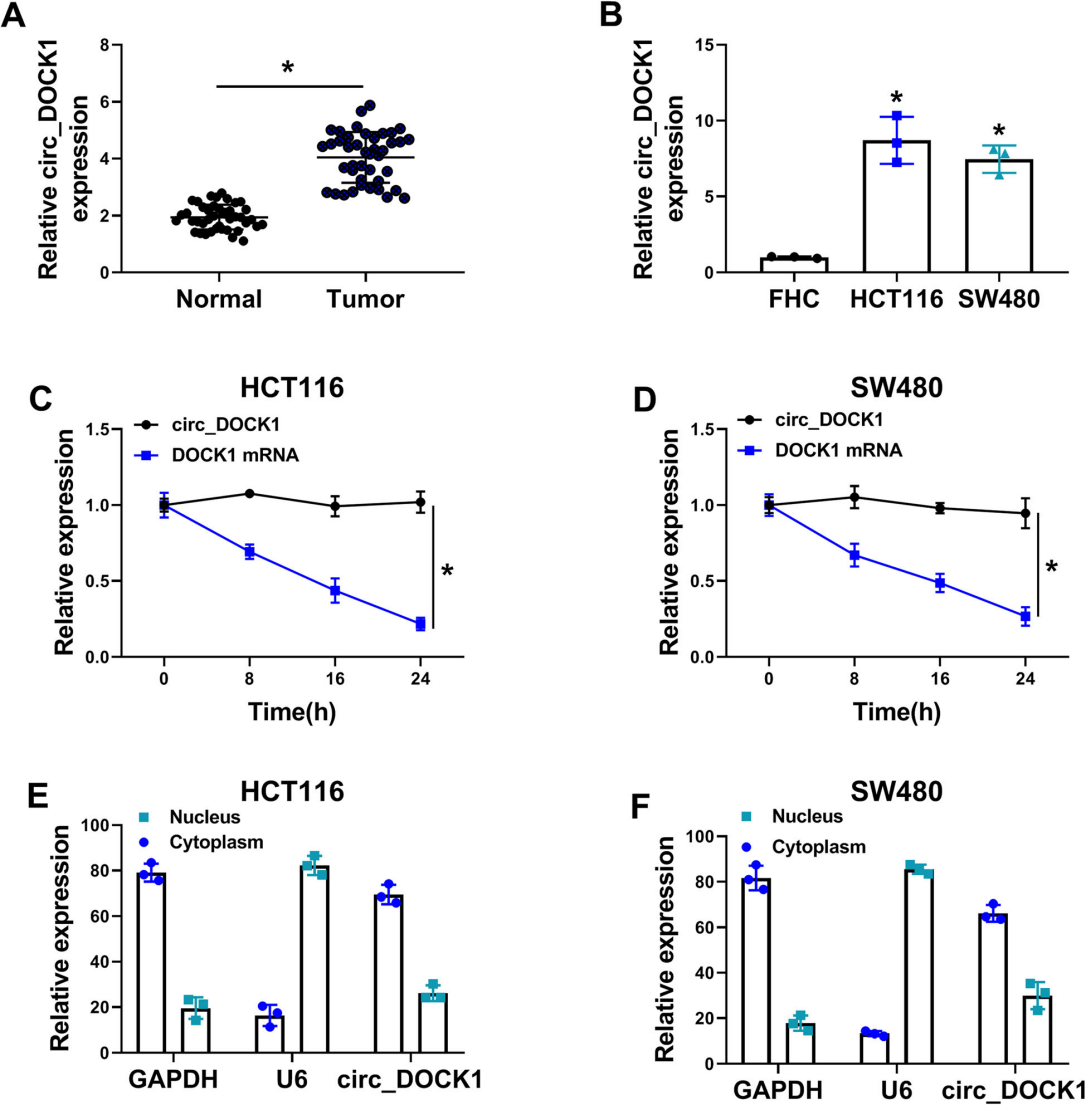
Circ_DOCK1 expression is enhanced in colorectal cancer samples and cell lines. **a** Circ_DOCK1 expression was determined using qRT-PCR in tumor and normal samples. *n*=42. **b** Circ_DOCK1 level was measured via qRT-PCR in HCT116, SW480, and FHC cells. **c**, **d** Circ_DOCK1 and DOCK1 levels were examined via qRT-PCR in HCT116 and SW480 cells after actinomycin D treatment for 0, 8, 16, or 24 h. **e**, **f** Circ_DOCK1, β-actin, and U6 levels were measured via qRT-PCR in nucleus and cytoplasm of HCT116 and SW480 cells. *P<0.05

To probe the role of circ_DOCK1 in colorectal cancer development, its expression was examined. By detecting circ_DOCK1 expression in 42 paired tumor and normal tissues, results showed circ_DOCK1 (hsa_circ_0020397) level was significantly increased in colorectal cancer tissues compared with normal samples (Fig. 1a), while hsa_circ_0020394 level was not obviously changed in tumor and normal samples (Supplementary Figure 1). Furthermore, higher level of circ_DOCK1 was displayed in colorectal cancer cell lines (HCT116 and SW480) than human normal colonic epithelial cell line FHC cells (Fig. 1b). Moreover, the circular structure of circ_DOCK1 was confirmed by actinomycin D (a transcriptional inhibitor) assay, which showed circ_DOCK1 was more resistant to actinomycin D treatment than DOCK1 mRNA (Fig. 1 c and d). Additionally, circ_DOCK1 abundance was detected in nucleus and cytoplasm of HCT116 and SW480 cells. Results showed circ_DOCK1 was mostly expressed in the cytoplasm (Fig. 1 e and f). These results suggested increased circ_DOCK1 might be associated with colorectal cancer development.

### Circ_DOCK1 knockdown inhibits cell growth and metastasis and triggers apoptosis in colorectal cancer

**Fig. 2:**
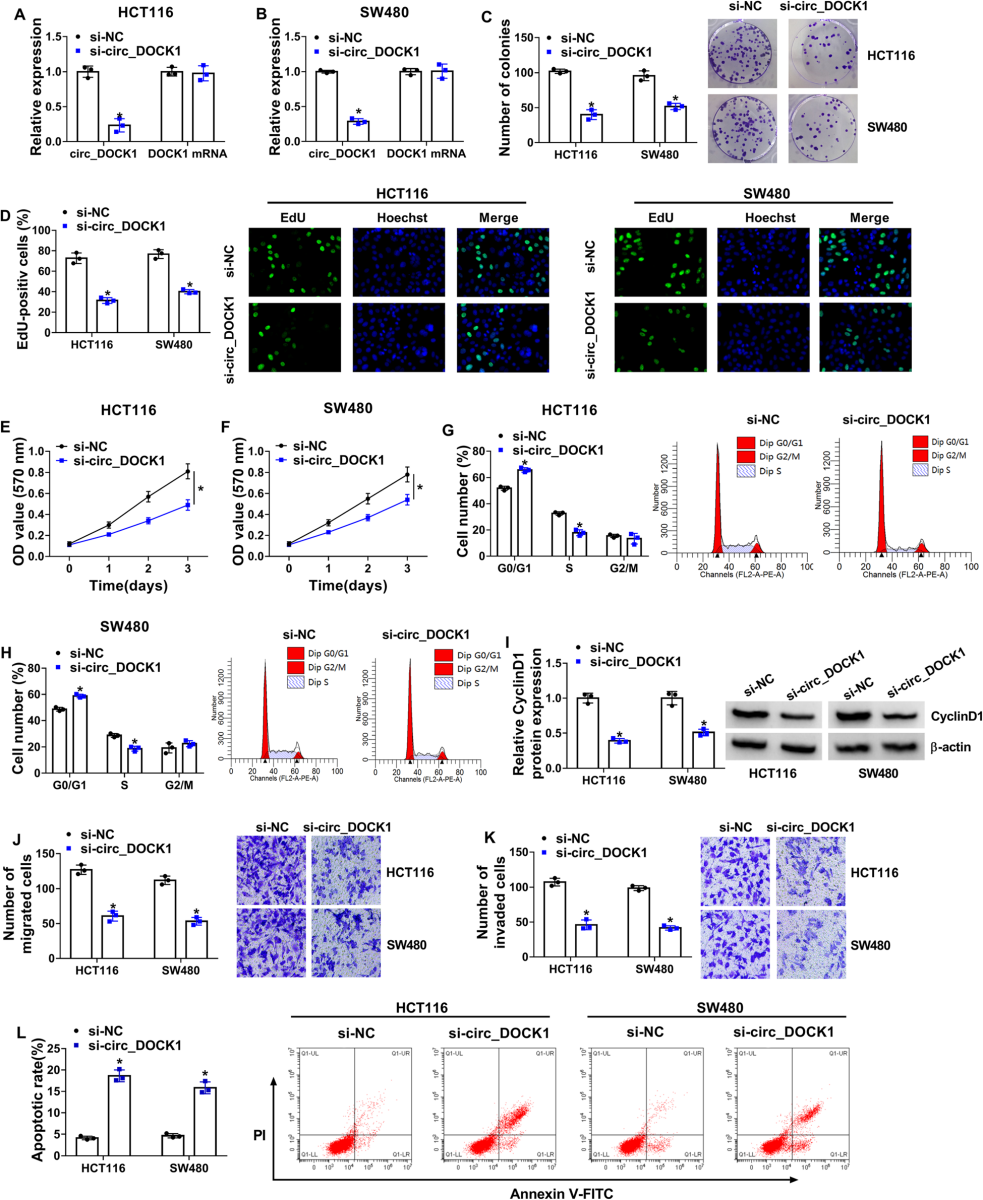
Circ_DOCK1 knockdown suppresses colorectal cancer cell progression. HCT116 and SW480 cells were transfected with si-circ_DOCK1 or si-NC. **a**, **b** Circ_DOCK1 and DOCK1 levels were measured using qRT-PCR in the transfected cells. **c** The colony-formation ability was examined via colony formation analysis in the transfected cells. **d**–**f** Cell proliferation was detected by EdU staining and MTT. **g**, **h** Cycle process was detected via flow cytometry. **i** CylinD1 expression was measured via Western blotting. **j**, **k** Cell migration and invasion were detected by transwell analysis. **l** Apoptosis was determined via flow cytometry. *P<0.05

To evaluate the influence of circ_DOCK1 on colorectal cancer development, HCT116 and SW480 cells were transfected with si-circ_DOCK1 or si-NC. The addition of si-circ_DOCK1 effectively reduced circ_DOCK1 expression but showed little influence on DOCK1 mRNA expression (Fig. 2 a and b). Moreover, cell growth was evaluated via colony formation, Edu staining, MTT, cycle distribution, and related protein expression. By detecting these events, results showed that circ_DOCK1 knockdown evidently reduced growth of HCT116 and SW480 cells by decreasing colony-formation ability, EdU-positive ratio, cell proliferation, and CyclinD1 expression, and inducing cycle arrest at G0/G1 phase (Fig. 2c–i). Additionally, metastasis was assessed by cell migration and invasion. Results displayed that circ_DOCK1 silence obviously inhibited the migratory and invasive abilities (Fig. 2 j and k). Furthermore, circ_DOCK1 interference led to clearly higher apoptotic production in HCT116 and SW480 cells (Fig. 2l). These data suggested silence of circ_DOCK1 could suppress colorectal cancer development.

### MiR-132-3p is targeted by circ_DOCK1 and downregulated in colorectal cancer

**Fig. 3:**
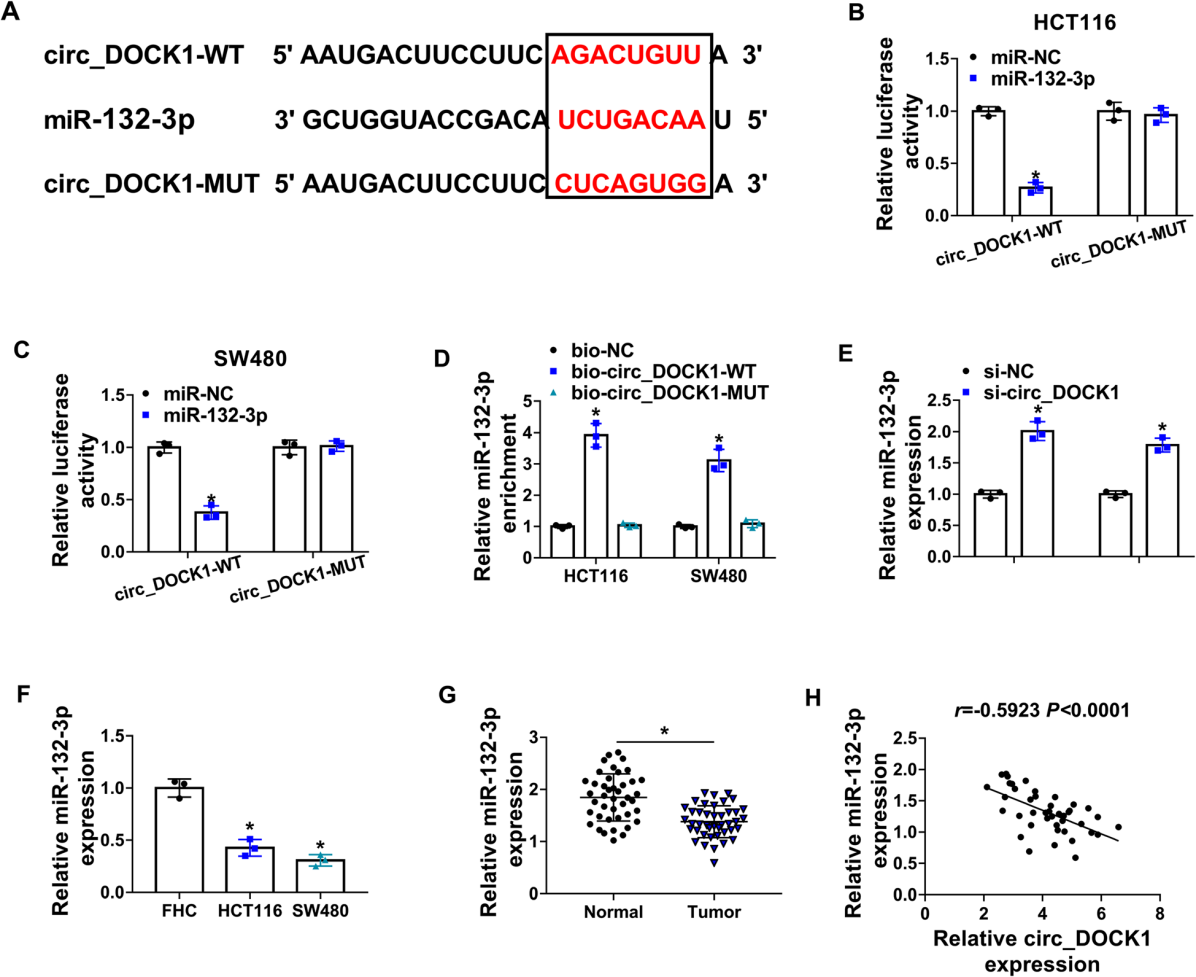
Circ_DOCK1 sponges miR-132-3p. **a** Predictive target sites of circ_DOCK1 and miR-132-5p were provided by circBank. **b**, **c** Luciferase activity was detected in cells transfected with miR-NC or miR-132-3p mimic and circ_DOCK1-WT or circ_DOCK1-MUT. **d** MiR-132-3p level was detected via qRT-PCR after RNA pull-down. **e** MiR-132-3p expression was detected via qRT-PCR in cells with transfection of si-NC or si-circ_DOCK1. **f** MiR-132-3p level was measured via qRT-PCR in HCT116, SW480, and FHC cells. **g** MiR-132-3p expression was detected via qRT-PCR in tumor and normal tissues. *n*=42. **h** The linear association of circ_DOCK1 and miR-132-3p abundances in tumor tissues. *P<0.05

To analyze the mechanism addressed via circ_DOCK1 in colorectal cancer, the targets of circ_DOCK1 were predicted via circBank. The target sites of circ_DOCK1 on miR-132-3p are shown in Fig. 3a. The circ_DOCK1-WT and circ_DOCK1-MUT vectors were constructed, and miR-132-3p mimic markedly inhibited luciferase activity of circ_DOCK1-WT but did not change the activity of circ_DOCK1-MUT (Fig. 3 b and c). Furthermore, RNA pull-down assay showed miR-132-3p could be bound to circ_DOCK1 in bio-circ_DOCK1-WT group, while the enrichment was abolished in bio-circ_DOCK1-MUT group (Fig. 3d). Additionally, miR-132-3p abundance was increased via circ_DOCK1 silence (Fig. 3e). Moreover, miR-132-3p abundance was evidently reduced in colorectal cancer cells and tumor tissues (Fig. 3 f and g). And it was inversely associated with circ_DOCK1 level in colorectal cancer (Fig. 3h). These results suggested miR-132-3p was sponged via circ_DOCK1.

### MiR-132-3p downregulation attenuates the influence of circ_DOCK1 interference on cell growth, metastasis, and apoptosis in colorectal cancer cells

**Fig. 4:**
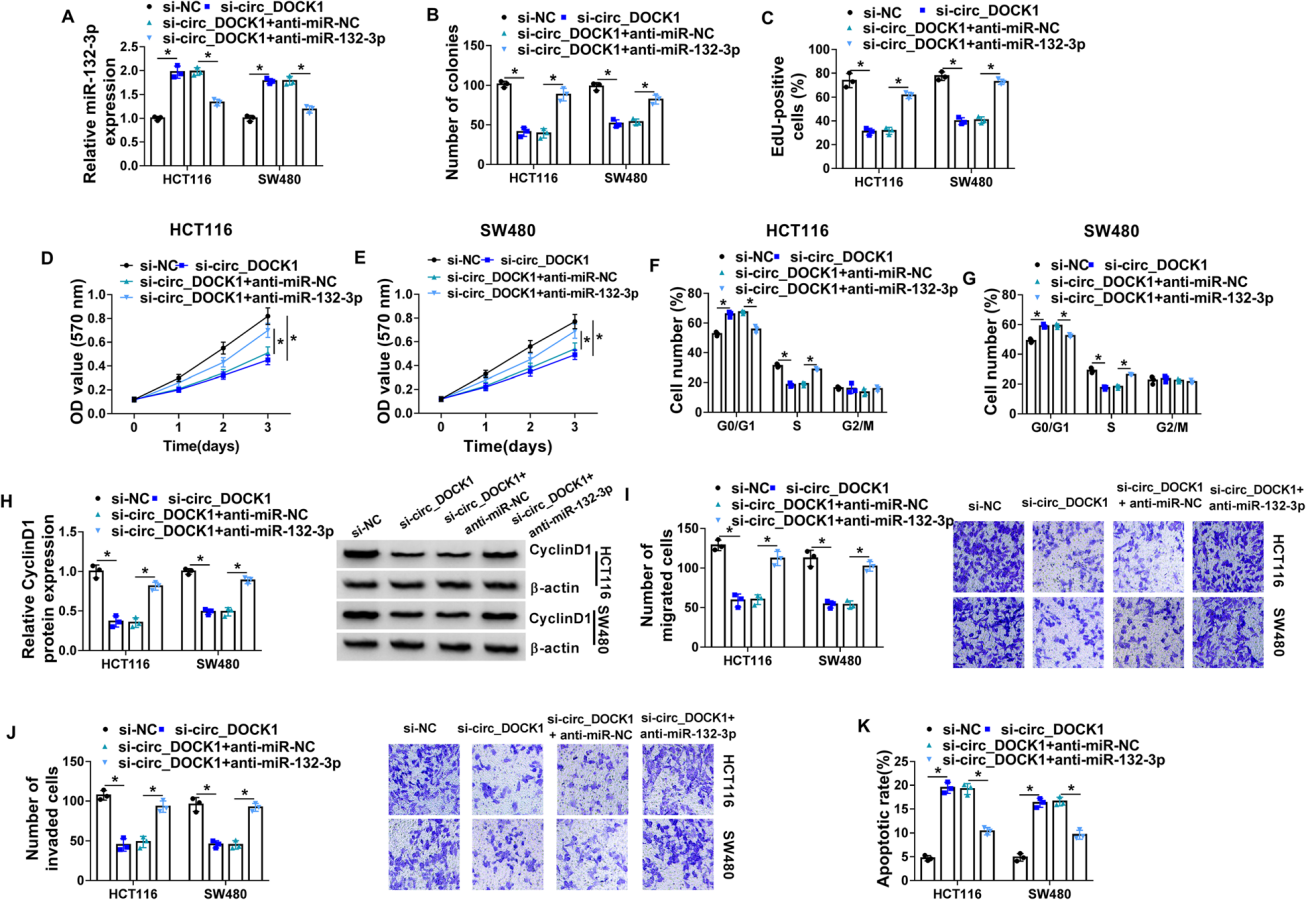
MiR-132-3p knockdown reverses silencing circ_DOCK1-modulated cell proliferation, migration, invasion, and apoptosis in colorectal cancer cell lines. HCT116 and SW480 cells were transfected with si-NC, si-circ_DOCK1, si-circ_DOCK1 + anti-miR-NC, or anti-miR-132-3p. MiR-132-3p expression (**a**), colony-formation ability (**b**), cell proliferation (**c**–**e**), cycle distribution (**f** and **g**), CyclinD1 expression (**h**), migration (**i**), invasion (**j**), and apoptosis (**k**) were detected via qRT-PCR, colony formation assay, EdU staining, MTT, flow cytometry, Western blotting, and transwell analysis in the transfected cells. *P<0.05

To investigate if miR-132-3p was involved in circ_DOCK1-mediated colorectal cancer progression, HCT116 and SW480 cells were transfected with si-NC, si-circ_DOCK1, si-circ_DOCK1 + anti-miR-NC, or anti-miR-132-3p. MiR-132-3p expression was markedly enhanced via si-circ_DOCK1, and it was reduced via addition of anti-miR-132-3p (Fig. 4a). Moreover, miR-132-3p downregulation cell growth by alleviating silence of circ_DOCK1-modulated loss of colony-formation ability, EdU-positive ratio, cell proliferation, and CyclinD1 expression, and promotion of cycle arrest at G0/G1 phase (Fig. 4b–h). Additionally, miR-132-3p knockdown mitigated interference of circ_DOCK1-modulated migration and invasion inhibition (Fig. 4 i and j). Furthermore, miR-132-3p inhibition weakened knockdown of circ_DOCK1-induced promotion of apoptosis in HCT116 and SW480 cells (Fig. 4k). These results indicated that knockdown of circ_DOCK1 could suppress colorectal cancer development via regulating miR-132-3p.

### USP11 is targeted by miR-132-3p and regulated via circ_DOCK1/miR-132-3p axis

**Fig. 5:**
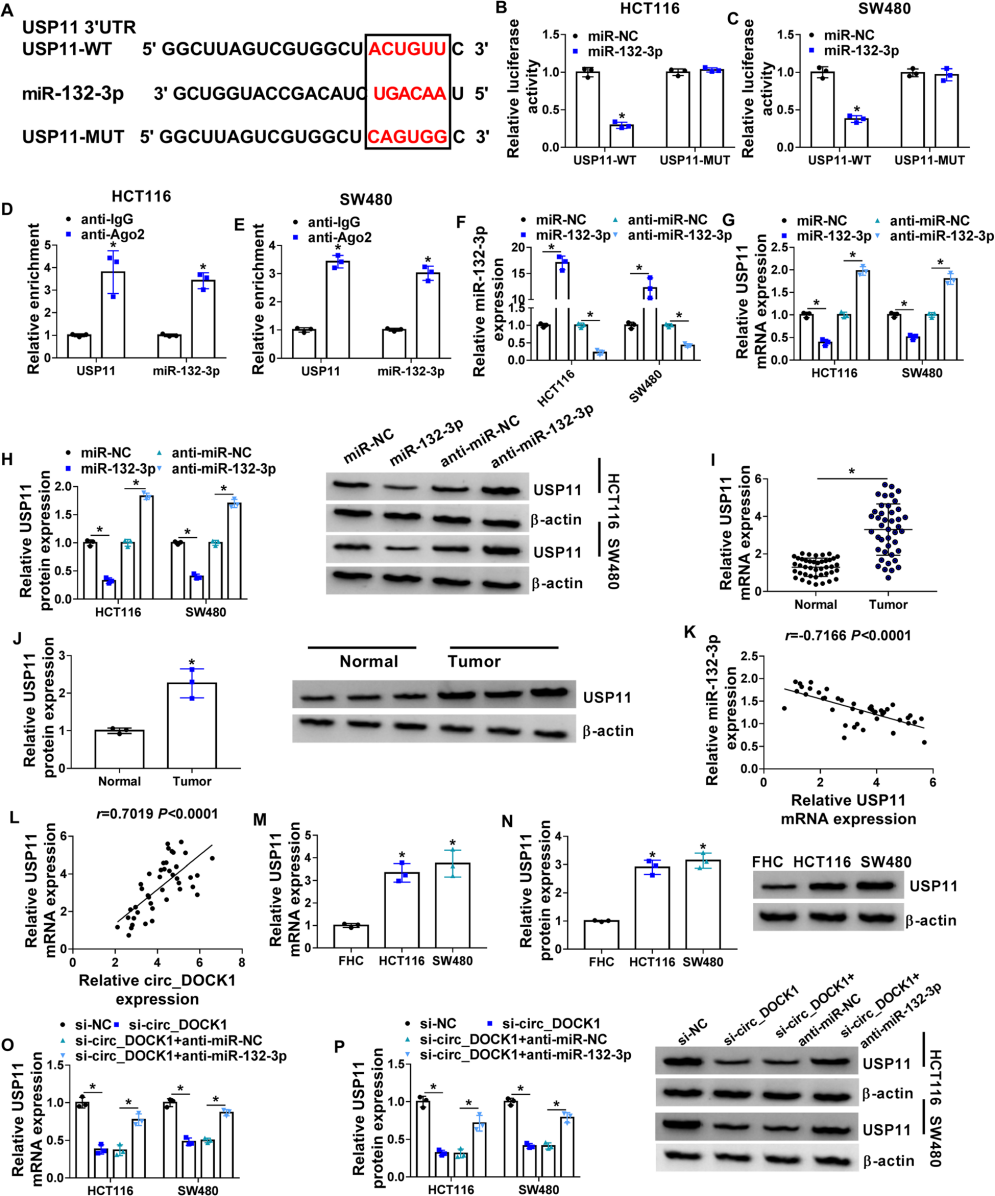
MiR-132-3p targets USP11. **a** The binding sites of miR-132-3p on USP11 were predicted by starBase. **b**, **c** Luciferase activity was detected in cells transfected with miR-NC or miR-132-3p mimic and USP11-WT or USP11-MUT. **d**, **e** USP11 and miR-132-3p levels were examined via qRT-PCR in cells after RIP. **f**–**h** MiR-132-3p and USP11 levels were measured via qRT-PCR or Western blotting in cells transfected with miR-NC, miR-132-3p mimic, anti-miR-NC, or anti-miR-132-3p. **i**, **j** USP11 expression was examined via qRT-PCR or Western blotting in tumor and normal tissues. **k**, **l** The linear association of USP11 and miR-132-3p or circ_DOCK1 levels in tumor tissues. **m**, **n** USP11 expression was determined via qRT-PCR or Western blotting in HCT116, SW480, and FHC cells. **o**, **p** USP11 level was determined via qRT-PCR or Western blotting in cells with transfection of si-NC, si-circ_DOCK1, si-circ_DOCK1 + anti-miR-NC, or anti-miR-132-3p. *P<0.05

To analyze the mechanism addressed by circ_DOCK1/miR-132-3p axis, the targets of miR-132-3p were predicted via starBase. The target sites of miR-132-3p on USP11 are exhibited in Fig. 5a. The USP11-WT and USP11-MUT vectors were constructed, and miR-132-3p mimic clearly inhibited the luciferase activity of USP11-WT, but it displayed little effect on activity of USP11-MUT (Fig. 5 b and c). Moreover, miR-132-3p USP11 and miR-132-3p might be enriched in the Ago2-based complex (Fig. 5 d and e). Then, the influence of miR-132-3p on USP11 level was assessed in HCT116 and SW480 cells. MiR-132-3p mimic or anti-miR-132-3p efficacy was validated in Fig. 5f, and USP11 expression was negatively regulated via miR-132-3p (Fig. 5 g and h). Furthermore, USP11 expression was markedly enhanced in colorectal cancer tissues (Fig. 5 i and j). And it was inversely associated with miR-132-3p level, but positively related to circ_DOCK1 level in colorectal cancer (Fig. 5 k and l). In addition, USP11 level was significantly higher in HCT116 and SW480 cells than FHC cells (Fig. 5 m and n). Besides, USP11 expression was markedly decreased via circ_DOCK1 silence, and it was restored by addition of anti-miR-132-3p (Fig. 5 o and p). Circ_DOCK1 could regulate SUP11 expression indirectly via miR-132-3p.

### MiR-132-3p constrains cell growth and metastasis and increases apoptosis by decreasing USP11 in colorectal cancer cells

**Fig. 6:**
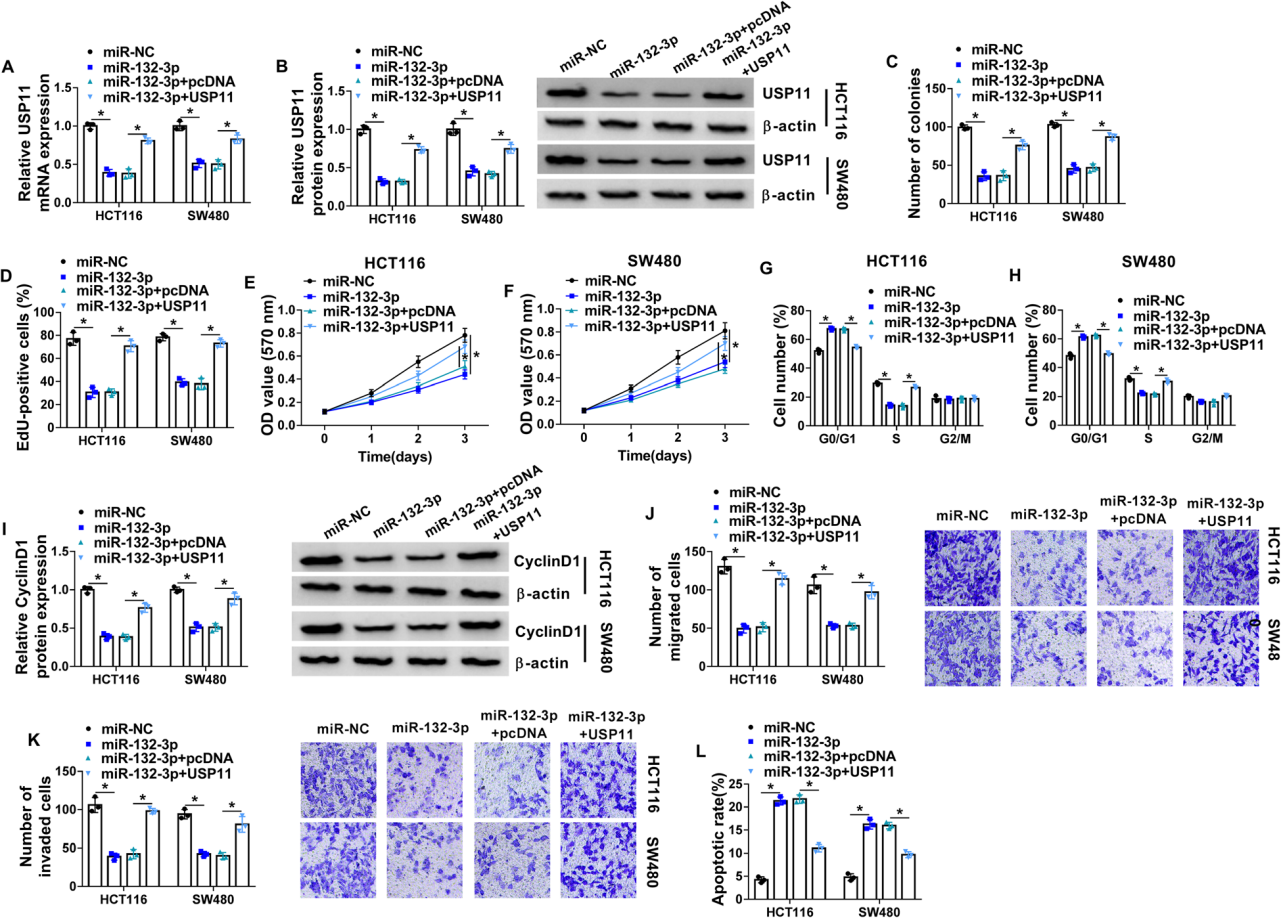
MiR-132-3p targets USP11 to suppress colorectal cancer progression. HCT116 and SW480 cells were transfected with miR-NC, miR-132-3p mimic, miR-132-3p mimic + pcDNA, or USP11 overexpression vector. USP11 expression (**a** and **b**), colony-formation ability (**c**), cell proliferation (**d**–**f**), cycle distribution (**g** and **h**), CyclinD1 expression (**i**), migration (**j**), invasion (**k**), and apoptosis (**l**) were examined via qRT-PCR, Western blotting, colony formation assay, EdU staining, MTT, flow cytometry, and transwell analysis in the transfected cells. *P<0.05

To study the function of miR-132-3p/USP11 axis on colorectal cancer progression, HCT116 and SW480 cells were transfected with miR-NC, miR-132-3p mimic, miR-132-3p mimic + pcDNA, or USP11 overexpression vector. USP11 abundance was significantly decreased by miR-132-3p overexpression, and it was upregulated via addition of USP11 overexpression vector (Fig. 6 a and b). Moreover, miR-132-3p overexpression evidently decreased cell growth by repressing the colony-formation ability, EdU-positive ratio, cell proliferation, and CyclinD1 expression, and increasing cycle arrest at G0/G1 phase, which were attenuated via USP11 upregulation (Fig. 6c–i). Additionally, miR-132-3p mimic obviously repressed cell migration and invasion in HCT116 and SW480 cells, and this effect was mitigated via addition of USP11 overexpression vector (Fig. 6 j and k). Furthermore, miR-132-3p mimic resulted in obvious apoptotic production in HCT116 and SW480 cells, and it was weakened via restoration of USP11 (Fig. 6l). These results suggested miR-132-3p overexpression could repress colorectal cancer development by targeting USP11.

### Circ_DOCK1 silence reduces tumor growth

**Fig. 7:**
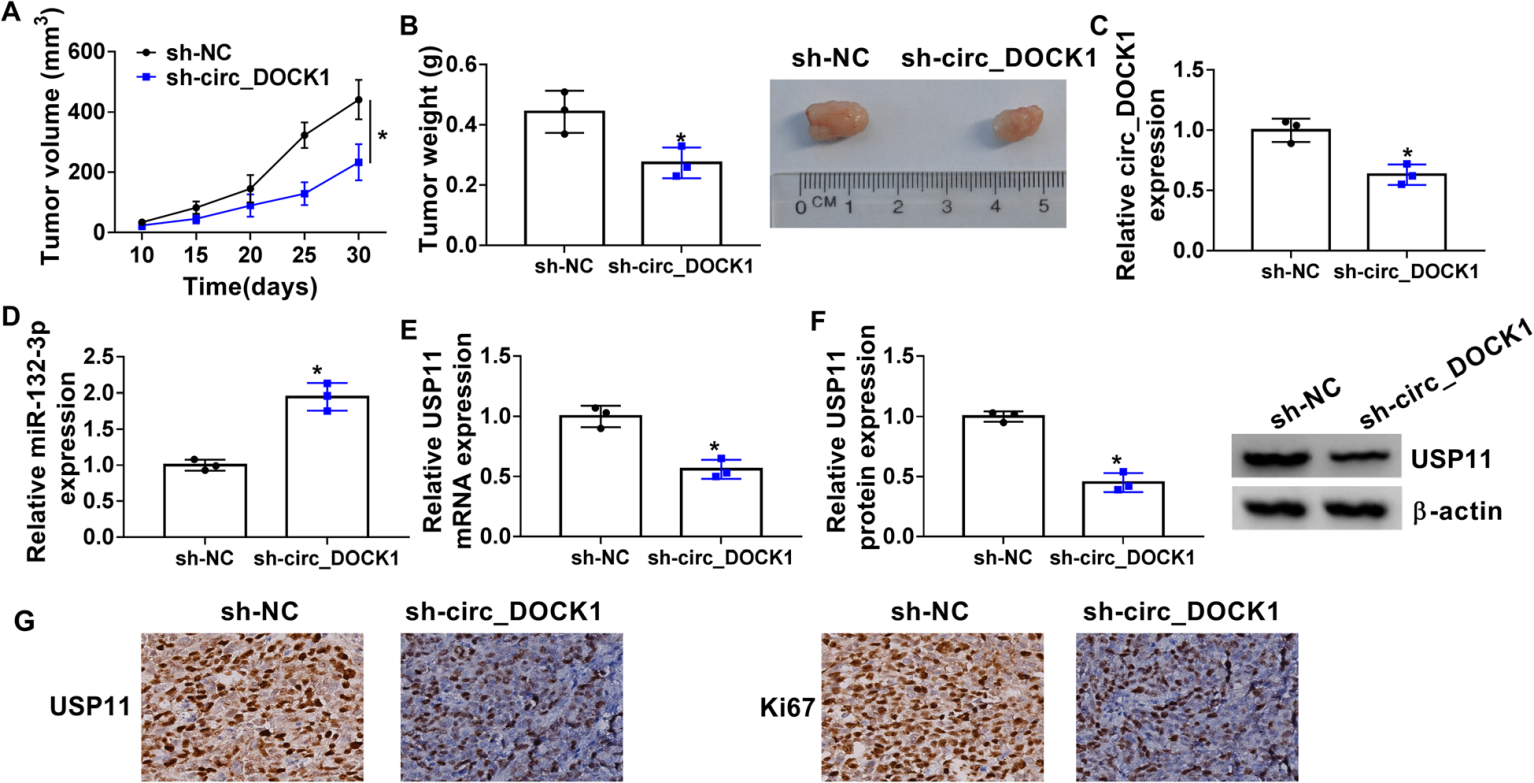
Circ_DOCK1 knockdown reduces cell growth in xenograft model. The sh-NC or sh-circ_DOCK1-transfetced HCT116 cells were subcutaneously injected in the nude mice, and mice were classified as sh-NC or sh-circ_DOCK1 group. *n*=5. **a**, **b** Tumor volume and weight were detected. **c**–**f** Circ_DOCK1, miR-132-3p, and USP11 levels were examined via qRT-PCR and Western blotting. **g** USP11 and Ki67 levels were measured via immunohistochemistry in tumor tissues. *P<0.05

**Fig. 8:**
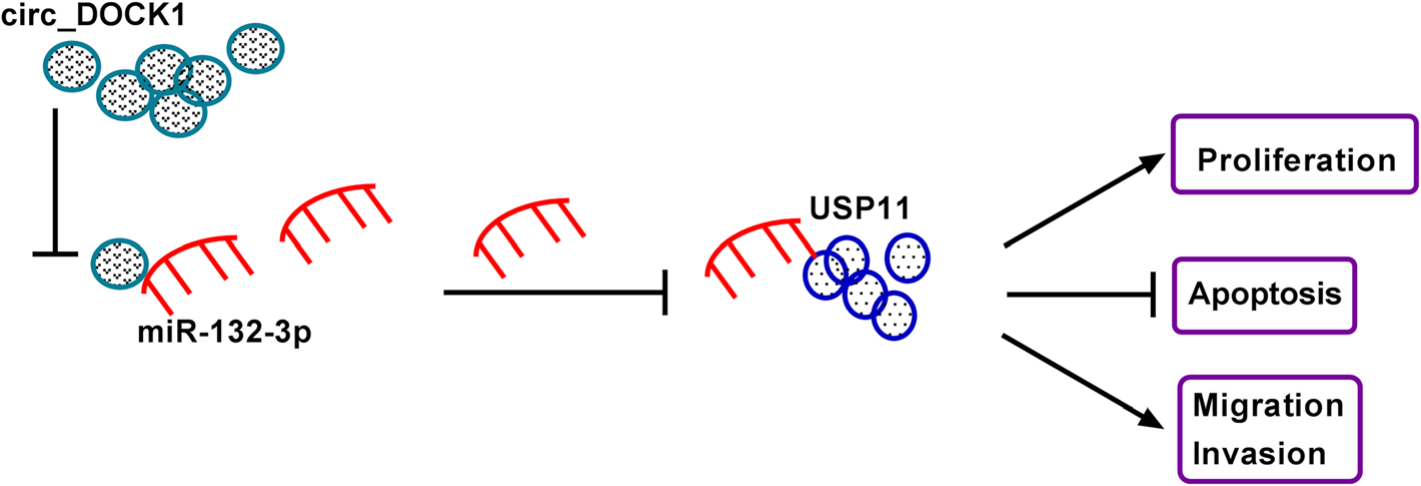
The schematic diagram of this study. Circ_DOCK1 sponges miR-132-3p to regulate USP11, thus increasing cell proliferation, migration, and invasion and suppressing apoptosis in colorectal cancer

To further probe the function of circ_DOCK1 in colorectal cancer, sh-circ_DOCK1- or sh-NC-transfected HCT116 cells were applied to establish the xenograft model. The mice were classified as sh-circ_DOCK1 or sh-NC group (*n*=5). Tumor volume and weight were markedly declined in the sh-circ_DOCK1 group in comparison to the sh-NC group (Fig. 7 a and b). In addition, circ_DOCK1 and USP11 abundances were clearly reduced, but miR-132-3p level was increased in the sh-circ_DOCK1 group compared with the sh-NC group (Fig. 7c–g). Additionally, the proliferation-related marker Ki67 expression was detected in tumor tissues. The data of immunohistochemistry showed Ki67 level was clearly lowered in the sh-circ_DOCK1 group (Fig. 7g). The schematic diagram of circ_DOCK1-drived mechanism is exhibited in Fig. 8, which showed that circ_DOCK1 regulated USP11 by sponging miR-132-3p, thus contributing to cell proliferation, migration, and invasion and repressing apoptosis in colorectal cancer.

## Discussion

Colorectal cancer is a common malignancy with high incidence and mortality [28]. Exploring new mechanism is helpful for finding novel strategy for colorectal cancer treatment. The noncoding RNAs like circRNAs have potential for the detection and therapy of colorectal cancer [29]. Our study evaluated the oncogenic function of circ_DOCK1 on colorectal cancer development, and we firstly confirmed the circ_DOCK1/miR-132-3p/USP11 axis.

Multiple isoforms of circ_DOCK1 have been reported to play important roles in colorectal cancer. For example, circ_DOCK1 (hsa_circ_0007142) facilitated cell proliferation, migration, and invasion by modulating miR-122-5p/cell division cycle 25A (CDC25A) in colorectal cancer [11]. Similarly, another isoform of circ_DOCK1 (hsa_circ_0020397) also modulated cell viability, apoptosis, and invasion through miR-138/telomerase reverse transcriptase (TERT)/programmed death-ligand 1 (PD-L1) axis in colorectal cancer [12]. These all suggested the oncogenic role of circ_DOCK1 in cancers. Here, we focused on the circ_DOCK1 (hsa_circ_0020397) isoform, and confirmed circ_DOCK1 knockdown could repress cell growth and metastasis, and promoted apoptosis in colorectal cancer, which suggested circ_DOCK1 might function as an important target for colorectal cancer treatment.

Next, we explored additional regulatory network except for circ_DOCK1/miR-138/TERT/PD-L1 axis in colorectal cancer [12]. Here we firstly confirmed circ_DOCK1 (hsa_circ_0020397) could sponge miR-132-3p, although a previous report has reported the interaction between miR-132-3p and the other isoform (hsa_circ_0020394) [10]. Song et al. reported miR-132-3p/mitogen-activated protein kinase 1 (MAPK1) axis might be regulated by X-inactive specific transcript (XIST) to suppress cell growth in colorectal cancer [30]. Furthermore, previous studies reported miR-132-3p restrained growth and metastasis of colorectal cancer cells via decreasing Derlin-1 or cAMP responsive element binding protein 5 (CREB5) [31, 32]. Additionally, Wang et al. suggested miR-132-3p inhibited cell growth and metastasis of resistant cells to regulate 5-fluorouracil (5-FU) resistance in colorectal cancer [33]. These reports suggested the anti-tumor role of miR-132-3p in colorectal cancer. Likewise, we also validated the anti-cancer function of this miRNA and found that circ_DOCK1 could regulate colorectal cancer progression by sponging miR-132-3p.

Then, we analyzed the downstream of miR-132-3p and firstly confirmed miR-132-3p could target USP11. USP11 acted as a common deubiquitinase that can promote cell growth via modulating cell cycle processes and DNA repair [22, 34]. Moreover, previous evidences suggested USP11 could promote epithelial-to-mesenchymal transition to increase cell metastasis in ovarian cancer and breast cancer [35, 36]. Additionally, USP11 could promote cell proliferation and metastasis via regulating nuclear factor 90 (NF90) in hepatocellular carcinoma [37]. More importantly, USP11 could promote cell growth and metastasis in colorectal cancer [23, 38]. These reports indicated the oncogenic role of USP11 in human tumors, including colorectal cancer. Consistent with these reports, our study found USP11 reversed the anti-cancer function of miR-132-3p, indicating miR-132-3p could modulate colorectal cancer development by targeting USP11. Furthermore, we found circ_DOCK1 regulated USP11 indirectly via miR-132-3p. In this way, the circ_DOCK1/miR-132-3p/USP11 axis participated in colorectal cancer development. However, the current work did not explore the downstream and specific function of USP11 in colorectal cancer, which would be explored in future.

In conclusion, our study displayed that circ_DOCK1 was upregulated in colorectal cancer, and circ_DOCK1 knockdown repressed growth and metastasis, and promoted apoptosis of colorectal cancer cells, possibly via increasing miR-132-3p and decreasing USP11. This study provided a new insight for understanding the mechanism of colorectal cancer and provided a target for colorectal cancer treatment.

## Supplementary Information

**Additional file 1: Supplementary Figure 1**. Hsa_circ_0020394 expression in colorectal cancer. Hsa_circ_0020394 level was detected by qRT-PCR in tumor and normal samples. n=42.

## Data Availability

The analyzed data sets generated during the present study are available from the corresponding author on reasonable request.
