# Supplementary figures and images for: Circ_DOCK1 regulates USP11 through miR-132-3p to control colorectal cancer progression
Source: World J Surg Oncol. 2021 Mar 8;19:67. doi: 10.1186/s12957-021-02173-x (PMC7941900; doi:10.1186/s12957-021-02173-x)

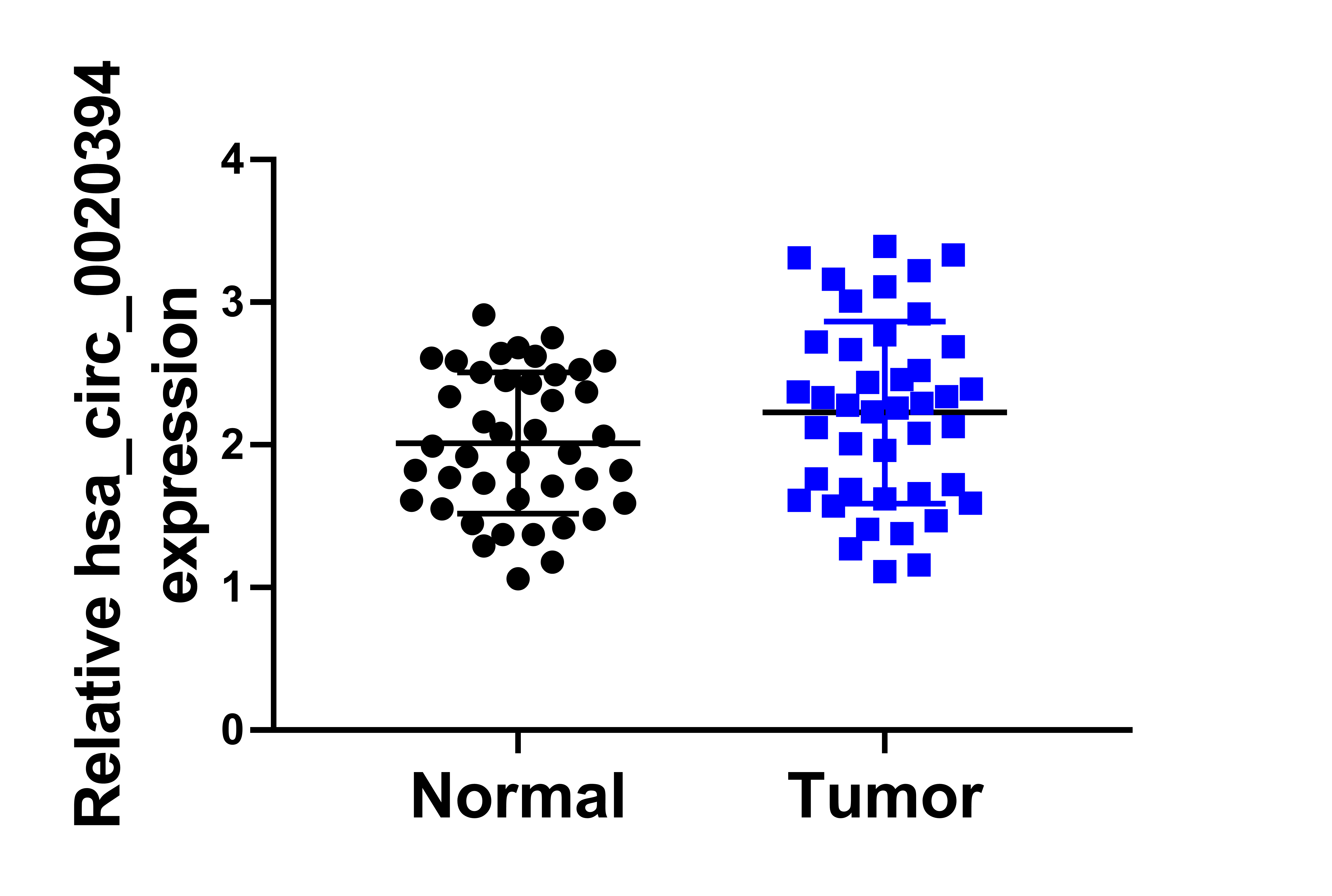

Supplement: Supplementary file 1 — Additional file 1: Supplementary Figure 1. Hsa_circ_0020394 expression in colorectal cancer. Hsa_circ_0020394 level was detected by qRT-PCR in tumor and normal samples. n=42. [file 12957_2021_2173_MOESM1_ESM.tif]
